# Anhedonia underlies the association between childhood unpredictability and adult PTSD symptoms: Evidence from three independent longitudinal cohorts

**DOI:** 10.1017/S0033291726103316

**Published:** 2026-03-05

**Authors:** Christopher Hunt, Laura M. Glynn, Elysia Poggi Davis, Tallie Z. Baram, Caroline M. Nievergelt, Giovanni Castillo, Christian Cortes, Christine Y. Chang, Dewleen Baker, Victoria B. Risbrough

**Affiliations:** 1 VA San Diego Healthcare System, San Diego, CA, USA; 2 Veteran Affairs Center of Excellence for Stress and Mental Health, La Jolla, CA, USA; 3 Psychiatry, University of California San Diego, La Jolla, CA, USA; 4 Psychology, Chapman University, Orange, CA, USA; 5 University of Denver, Denver, CO, USA; 6 Pediatrics, University of California Irvine, Irvine, CA, USA; 7 Pediatrics, Anatomy/Neurobiology, Neurology University of California Irvine, Irvine, CA, USA; 8 Psychology, University of Washington, Seattle, WA, USA

**Keywords:** anhedonia, early life adversity, prospective longitudinal, PTSD, reward, trauma, unpredictability

## Abstract

**Background:**

Unpredictability in the child’s environment has recently emerged as a significant and unique form of early life adversity (ELA). Cross-sectional studies have linked childhood unpredictability with increased post-traumatic stress disorder (PTSD) symptoms in adults; however, no prospective studies have tested the link between childhood unpredictability and PTSD risk in later life, nor what processes, such as increased anhedonia symptoms, might mediate such risk. Here, we leveraged three distinct prospective, longitudinal cohorts to test the hypothesis that unpredictability during childhood contributes to adult PTSD via worsening anhedonia symptoms.

**Methods:**

Participants were male service members (*n*=314), adult females (*n*=170), and adolescents (*n*=137) recruited for separate longitudinal investigations. All completed dimensional assessments of anhedonia symptoms and PTSD; childhood trauma and childhood unpredictability were measured by the Questionnaire for Unpredictability in Childhood (QUIC). Pearson correlations tested relations between QUIC, anhedonia symptoms, and PTSD symptoms. Mediational models tested whether the link between childhood unpredictability and PTSD is mediated by increased anhedonia symptoms by estimating indirect effects via bootstrapped path analysis.

**Results:**

Childhood unpredictability was associated with increased adult PTSD symptoms in all three cohorts (*r*s>.19, *p*s<.016). Further, in all three cohorts, the relationship was partially mediated by higher anhedonia symptoms (*bs*>0.046, 95% confidence intervals = 0.01–0.12). All effects remained significant when controlling for levels of childhood trauma and removing anhedonia-related PTSD items.

**Conclusions:**

Unpredictability during childhood may confer risk for adult PTSD, and this increased risk may occur via alterations in anhedonia symptoms. Efforts to increase predictability during childhood could enhance resilience to later traumatic events.

## Introduction

Childhood adversity, including childhood trauma, abuse, and neglect, are established contributors to the risk for the development of mental health disorders, including posttraumatic stress disorder (PTSD; Kessler et al., 2017). Accumulating evidence suggests that *unpredictability* in the child’s caregiving and environment can also have long-term consequences for mental health (von Stumm, 2024; Keen et al., 2023; Spadoni et al., 2022; Aran et al., 2024), independent of other forms of adversity (e.g. abuse, neglect, poverty, Spadoni et al., 2022; poverty: Keen et al., 2023). Unpredictability of parental and environmental signals is characterized by lability of caregiver behavior and mood in addition to a disrupted home environment (e.g. frequent moves, lack of household routine). The notion that unpredictability acts as a potent form of ELA is grounded in various neurodevelopmental theories, including life-history theory (adaptation to developmental context; Ellis, Sheridan, Belsky, & McLaughlin, 2022), attachment theory (emphasizes a consistent and responsive caregiver; Sroufe, 2005), theories related to household chaos/family routine (Marsh, Dobson, & Maddison, 2020), learning theory (predictability in sensory input and action consequences; Köster, Kayhan, Langeloh, & Hoehl, 2020), as well as biological evidence that inconsistent patterns of caregiver signals can shape neural circuit formation and synaptic connectivity during development (Birnie & Baram, 2022). In the last decade, unpredictability in early life has emerged as a unique contributor to emotional and cognitive development, leading to a more comprehensive understanding of early-life adversity mechanisms, as well as policy and intervention strategies to mitigate its effects (Glynn, 2025; Baram et al., 2012).

One mechanism through which early-life unpredictability may shape adult psychopathology is via modification of the reward system. In rodents, unpredictable maternal behaviors disrupt reward circuit connectivity and increase anhedonia-like phenotypes, operationalized by lowered reward sensitivity and reduced pleasure-seeking behavior (Birnie et al., 2023; Bolton et al., 2018; Kangas et al., 2022; Levis et al., 2022). In humans, unpredictability in early life is linked to aberrant connectivity and maturation in circuits related to reward processing and response (Glynn & Baram, 2019; Short & Baram, 2019; Granger et al., 2021; Wang et al., 2024). Given this extensive cross-species evidence, it is reasonable to suspect that unpredictability in childhood might confer risk for psychiatric symptoms related to the reward function – such as anhedonia. Anhedonia (or hypohedonia) is the reduced capacity to experience pleasure or interest in typically rewarding experiences and often reflects disruptions in the brain’s reward systems (Der-Avakian & Markou, 2012; Treadway & Zald, 2011). Rather than a simple absence of positive affect, anhedonia encompasses broader impairments in how rewards are processed, anticipated, and valued – processes that may be particularly sensitive to unpredictable sensory signals from the caregiver and environment (Pizzagalli, 2014; Whitton, Treadway, & Pizzagalli, 2015). Both cross-sectional and prospective studies have linked anhedonia symptoms in adulthood to childhood unpredictability (Spadoni et al., 2022; Null et al., 2024; Hunt et al., 2024); however, no prospective studies have shown if increased anhedonia symptoms mediate the contribution of early life unpredictability to adult neuropsychiatric disorders.

Extant hypotheses suggest that PTSD symptoms may partly stem from reduced motivation for reward, in addition to the threat hyperresponsiveness traditionally linked to the disorder. During motivational conflict, reduced reward processing and/or responses could bias behavioral decision-making toward avoidance, even at the risk of loss of reward (Akiki et al., 2025; Bennett, Davis, & Fitzgerald, 2023). PTSD is consistently associated with reward-system deficits, including disruption in reward processing/circuits and comorbid anhedonia symptoms, with recent evidence suggesting that these deficits contribute to both development and maintenance of PTSD (Risbrough et al., 2018; Admon et al., 2013). Acute reward disruption post-trauma is associated with risk for long-term PTSD (Stevens et al., 2021), while intact reward behavior may be a resiliency factor for fear-based disorders like PTSD (Kalisch, Russo, & Müller, 2024). Reward circuit hyporesponsivity and self-reported anhedonia *prior* to trauma exposure also predict the development and maintenance of PTSD symptoms post-trauma (Acheson et al., 2022; Admon et al., 2013). Cross-sectional analyses indicate that anhedonia mediates childhood trauma associations with PTSD (Haim-Nachum et al., 2024), supporting the idea that reward disruption, as measured by anhedonia, may mediate the increased risk for PTSD associated with ELA, including unpredictability. However, no prospective, longitudinal studies have investigated the effect of childhood unpredictability on the development of PTSD symptoms, nor the putative role of anhedonia symptoms as a mediating factor.

This study used a prospective, longitudinal design to test the hypotheses that (1) childhood unpredictability acts as a risk factor for adult PTSD symptoms, and (2) the risk associated with childhood unpredictability is conferred via decreased reward processes as measured by higher anhedonia symptoms. These hypotheses were investigated using longitudinal data on childhood unpredictability, anhedonia symptoms, and PTSD from three distinct cohorts: (1) Adult male service members, (2) adult females, and (3) male and female adolescents. As such, the present study sought to examine a mechanism through which childhood unpredictability might lead to adult PTSD symptoms and determine the extent to which this mechanism is generalizable across development and different populations. We hypothesized that the link between higher unpredictability during childhood and higher PTSD symptoms later in adulthood would be partially mediated by higher anhedonia symptoms earlier in life, and that both the main effect of unpredictability and the mediating effect of anhedonia symptoms would be present in each cohort. Overall, the present study should advance our understanding of early life risk factors for PTSD symptoms later in life.

## Method

### Participants and procedures

#### Male service member cohort

The service member cohort consisted of 314 active-duty service members or Veterans who had participated in the Marine Resiliency Study (Baker et al., 2012), a longitudinal study of Marines and Navy Corpsman assessed prior to and following combat deployment. All study procedures were approved by the VA San Diego Healthcare System and the University of California, San Diego, Institutional Review Boards. Participants provided written informed consent.

#### Adult female cohort

The cohort of adult females consisted of a community sample of 170 women participating in an ongoing, longitudinal study of maternal and child health recruited from Southern California medical clinics. All study procedures were approved by the responsible Human Subjects Review Board, and participants provided written informed consent.

#### Adolescent cohort

The adolescent cohort consisted of 137 male and female adolescents from an ongoing longitudinal study of early life influences on child and adolescent development. These adolescents have been followed prospectively from the prenatal period through adolescence (details described in Glynn et al., 2019). All study procedures were approved by the responsible Human Subjects Review Board. Individuals over 18 provided consent for themselves, and mothers provided consent for their children if they were younger than 18; participants younger than 18 also provided assent to participate.

All procedures contributing to this work comply with the ethical standards of the relevant national and institutional committees on human experimentation and with the Helsinki Declaration of 1975, as revised in 2008.

### Measures

**Figure 1.**
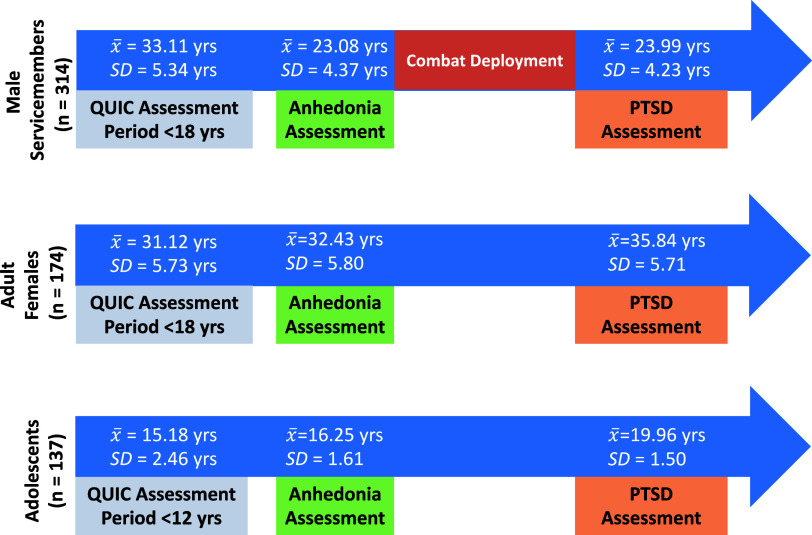
Timeline of measure administrations across the male service member (top), adult female (middle), and adolescent (bottom) cohorts. The period for the QUIC refers to the time period respondents were asked to consider when answering questions about childhood unpredictability. Anhedonia symptoms and PTSD assessments pertained to current symptoms. 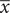 and *SD* refer, respectively, to the mean age and standard deviation of age at the time of administration for each measure, both of which inquired about current symptoms. For the male service member cohort, all anhedonia assessments were given prior to combat deployment, and all PTSD assessments were given following combat deployment. *Note:* QUIC = Questionnaire of Unpredictability in Childhood.

A timeline of childhood unpredictability, anhedonia symptoms, and PTSD assessments for each cohort, including the mean age of administration for each measure in each cohort, can be found in Figure 1. Gender, race, and ethnicity were self-reported by participants.

#### Childhood unpredictability

Childhood unpredictability was assessed with the Questionnaire of Unpredictability in Childhood (QUIC; Glynn et al., 2019), a 38-item retrospective self-report indexing social, emotional, and physical unpredictability during early life. Example items include ‘I experienced changes in my custody arrangement’ and ‘I usually knew when my parents were going to be home’. Nine items pertained to situations experienced prior to age 12, while 29 pertained to experiences prior to age 18. Higher scores indicated greater unpredictability during childhood. Across the three cohorts, the QUIC demonstrated excellent internal consistency (α ≈ .84–.92) and test–retest reliability (r ≈ .92). Evidence of construct validity includes prospective associations with objectively measured maternal unpredictability in infancy and concurrent associations with self-reported levels of household chaos, inconsistent discipline, and lax parental supervision during adolescence (Glynn et al., 2019). Importantly, the QUIC demonstrates unique associations with mental health symptoms after controlling for other measures of childhood adversity, such as income-to-needs ratio and childhood trauma, suggesting it is a unique dimension of ELA (Glynn et al., 2019). The QUIC is composed of five subscales: Parental Monitoring & Involvement, Parental Predictability, Parental Environment, Physical Environment, and Safety/Security. However, extant data do not support divergent associations with psychiatric symptoms for these subscales, and we had no specific hypotheses about subscales; therefore, only the total QUIC score was analyzed for this study.

#### Trauma exposure during childhood

The extent of childhood trauma was measured using the life events checklist (LEC; Weathers, Blake et al., 2013), a self-report inventory cataloging 16 discrete categories of potentially traumatic events (e.g. serious accidents, physical assault, sexual assault) and one ‘other’ category. Respondents indicate whether: (a) the event happened directly to them; (b) they witnessed the event happen to someone else; (c) they learned about the event happening to someone else; (d) it does not apply; or (e) they are not sure. In addition, for each endorsed event, participants indicated the earliest age at which the event occurred. For the adult cohorts, LEC total score was defined as the number of events endorsed as having *happened to them* or *witnessed* prior to the age of 18. For the adolescent cohort, LEC total was computed in the same way, but only LEC events that occurred prior to or concurrent with the QUIC were included in the total score to help ensure the trauma was not the result of unpredictability.

#### Anhedonia

##### Service member cohort

Anhedonia symptoms in the servicemember cohort were assessed using the Beck Depression Inventory anhedonia item subset (BDI-A). The BDI-A is composed of the three anhedonia-related items taken from the 21-item Beck Depression Inventory II (BDI-II; Beck et al., 1996), which is a self-report measure of depressive symptoms from the past 2 weeks. The BDI-A items on the BDI-II are: loss of pleasure, loss of interest, and loss of interest in sex. The ability of these three items to validly measure anhedonia symptoms was previously demonstrated in a principal component analysis using the same male service member cohort (see Acheson et al., 2022). Additionally, the BDI-A exhibits evidence of very good internal consistency reliability in this cohort across multiple assessments (α ≈ .74–.84; Hunt et al., 2024). Higher scores on the BDI-A reflect higher anhedonia symptom severity.

##### Adult female and adolescent cohorts

Anhedonia symptoms within the adult female and adolescent cohorts were assessed using the mood and anxiety symptom anhedonic-depression questionnaire (MASQ-AD; Clark & Watson, 1991). The MASQ-AD is a 22-item self-report subscale of the MASQ that assesses anhedonic depression symptoms over the past week. The MASQ-AD has demonstrated excellent internal consistency reliability across multiple samples (α ≈ .88–.96; Buckby et al., 2007; Talkovsky & Norton, 2015) and construct validity as demonstrated by good utility for identifying depressive disorders distinct from anxiety disorders (Buckby et al., 2007). Factor-analytic studies indicate that the MASQ-AD captures two correlated facets – loss of interest and low positive affect (Naragon-Gainey et al., 2009) – but given their conceptual overlap and the strong internal consistency of the total scale, we analyzed the composite score as a single indicator of anhedonia symptoms. Higher scores on this subscale indicate higher levels of anhedonia symptoms.

#### PTSD symptoms

##### Service member cohort

PTSD symptoms in service members were assessed using the Clinician Administered PTSD Scale for DSM-IV (CAPS-IV; Blake et al., 1995). The CAPS-IV is a semi-structured interview assessing the severity and frequency of the 17 diagnostic criteria for PTSD from the DSM-IV. The CAPS-IV yields a current or lifetime PTSD diagnosis and a total PTSD severity score. The CAPS-IV has demonstrated excellent psychometric properties, including excellent convergent validity with other measures of PTSD (Blake et al., 1995). A subset of the interviews was independently rerated by a second study clinician. Interrater reliability between interviewers for total CAPS-IV scores was excellent (ICC = 0.99) (Yurgil et al., 2014). Of note, CAPS-IV was utilized in the service member cohort because the study predated the release of DSM-5.

##### Adult female and adolescent cohorts

PTSD symptoms in the adult female and adolescent cohorts were assessed using the PTSD Checklist for DSM-5 (PCL-5; Weathers, et al., 2013), a 20-item self-report measure of PTSD symptoms that maps directly onto DSM-5 diagnostic criteria for PTSD. The PCL-5 has been shown to possess excellent psychometric properties across multiple populations (Blevins et al., 2015), including excellent internal consistency (α ≈ .84–.92) and test–retest reliability (*r* = .82), as well as strong convergent validity as demonstrated by high positive correlations with PTSD interview measures (e.g. CAPS-5). Higher scores indicate greater severity of PTSD symptoms.

### Analytical plan

#### Main analyses

Within each cohort, Pearson correlations tested associations among the main study variables. Correlations were used to confirm that (a) there was a zero-order relationship between QUIC and PTSD symptoms that mediation might explain and (b) the proposed mediator (anhedonia symptoms) is significantly associated with both the predictor (QUIC) and outcome (PTSD symptoms) variables, as is required by the selected mediation analysis approach (MacKinnon et al., 2002). If correlations were demonstrated, we next ran a hierarchical regression model with PTSD symptoms as the outcome variable, QUIC as a predictor in the first step, and anhedonia symptoms as a predictor in the second step. These models tested if including anhedonia symptoms in the model led to a reduction in the QUIC effect size – evidence of mediation according to our selected approach (MacKinnon et al., 2002). For models that met the above criteria, we then conducted a formal mediation analysis using model four of the SPSS PROCESS Macro (Preacher & Hayes, 2004). This model estimates direct and indirect effects using ordinary least squares path analysis. Indirect effects were tested with 10,000 bootstrap resamples to generate bias-corrected 95% confidence intervals (95% CIs), which are considered significant when they do not contain zero. This approach allows for inference about mediation effects without assuming normality of the indirect path distribution.

#### Sensitivity analyses

We ran three sensitivity analyses to address alternative explanations for study findings. First, we tested if effects of QUIC on PTSD and the mediating effect of anhedonia symptoms were independent of exposure to trauma in childhood. We tested this by including childhood LEC (first step) in a regression model in which QUIC (second step) was entered as a predictor of PTSD symptoms in each cohort and by rerunning the PROCESS mediation model, including childhood LEC as a covariate. Second, we reran all significant mediation models removing PTSD criterion D symptoms (i.e. seven items) from the PTSD total score, given that the content of criterion D overlaps significantly with anhedonia. Thus, this analysis was conducted to ensure that any mediating effect of anhedonia symptoms was not merely driven by symptom overlap with PTSD. Finally, for the service member cohort only, we reran the mediation controlling for PTSD symptoms at pre-deployment to verify that the mediating effect of anhedonia symptoms was not driven by pre-existing PTSD symptoms. Since PTSD symptoms were assessed at only one timepoint in the other two cohorts, this follow-up analysis could not be performed in those cohorts.

All analyses were conducted in SPSS version 28. Alpha was set at .05 (two-tailed) for all applicable tests.

## Results

### Sample characteristics

Demographic and clinical characteristics of each cohort can be found in Table 1.

**Table 1.** Demographic and clinical characteristics of the study samples

	Male Service members	Adult females	Adolescents
N	314	174	137
Gender			
% Male	100	0	46
% Female	0	100	54
Ethnicity			
% Black/African American	3.8	3.4	2.9
% Native American	1.6	0.0	0
% Asian	3.2	11.5	7.3
% Pacific Islander	0.6	0	0
% Hispanic/Latino	18.5	47.1	32.1
% White/Caucasian	65.0	31.6	44.5
% Multiple ethnicities	4.7	6.3	13.1
Mean Childhood LEC (SD)	2.47 (2.03)	1.16 (1.56)	1.95 (2.09)
Mean QUIC (SD)	10.39 (8.60)	7.77 (7.10)	6.80 (5.51)
Anhedonia measure	BDI-A	MASQ-AD	MASQ-AD
Anhedonia measure range	0–9	22–110	22–110
Mean anhedonia score (SD)	0.82 (1.28)	55.10 (14.59)	59.29 (14.15)
PTSD symptom measure	CAPS-IV	PCL–5	PCL–5
PTSD measure range	0–68	0–80	0–80
Mean PTSD symptom score (SD)	19.21 (19.14)	16.25 (15.58)	18.61 (16.29)

*Note:* Ranges refer to the range of possible values for the measure. Childhood LEC refers only to traumatic events that were directly experienced or witnessed during childhood; for the adolescent cohort, childhood LEC events were only those that were concurrent with or prior to the QUIC. QUIC = Questionnaire of Unpredictability in Childhood; LEC = Life events checklist; BDI-A = Beck Depression Inventory Anhedonia Items; MASQ-AD = Mood and anxiety symptom questionnaire anhedonic-depression subscale; CAPS-IV = Clinician Administered PTSD Scale for DSM-IV; PCL-5 = PTSD Checklist for DSM-5.

### Associations between primary study variables

**Figure 2.**
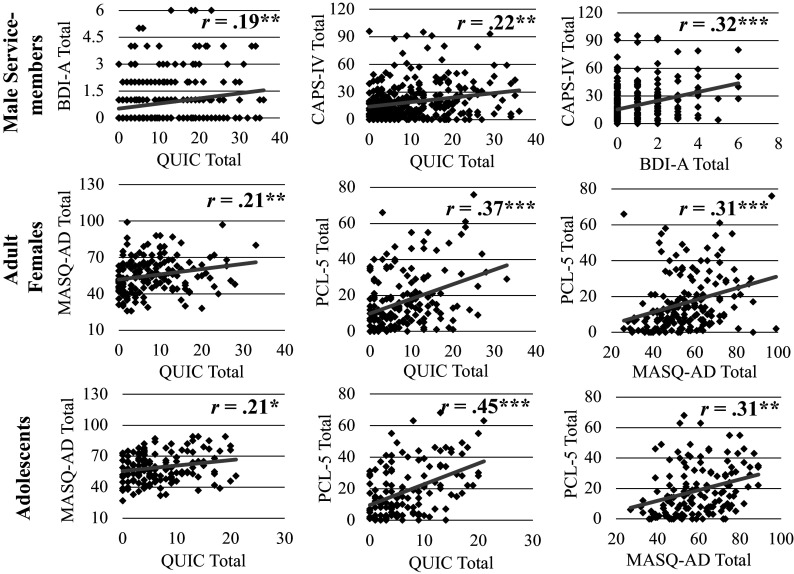
Scatterplots of associations between childhood unpredictability and anhedonia symptoms (left), childhood unpredictability and PTSD symptoms (middle), and anhedonia symptoms and PTSD symptoms (right) across cohorts of service members (top), adult females (middle), and adolescents (bottom). *Note:* QUIC = Questionnaire of Unpredictability in Childhood; BDI-A = Beck Depression Inventory Anhedonia Items; MASQ-AD = Mood and anxiety symptom questionnaire anhedonia subscale; CAPS-IV = Clinician Administered PTSD Scale for DSM-IV; PCL-5 = PTSD Checklist for DSM-5. ***p < .001; **p < .01; *p < .05.

Scatterplots of Pearson correlations between childhood unpredictability, PTSD symptoms, and anhedonia symptoms for each cohort are shown in Figure 2. Significant positive associations were observed for each of the three pairs of variables across all three cohorts (*r*s > .19, *p*s < .016).

### Testing anhedonia symptoms as a mediator of the longitudinal relationship between childhood unpredictability and PTSD symptoms

**Figure 3.**
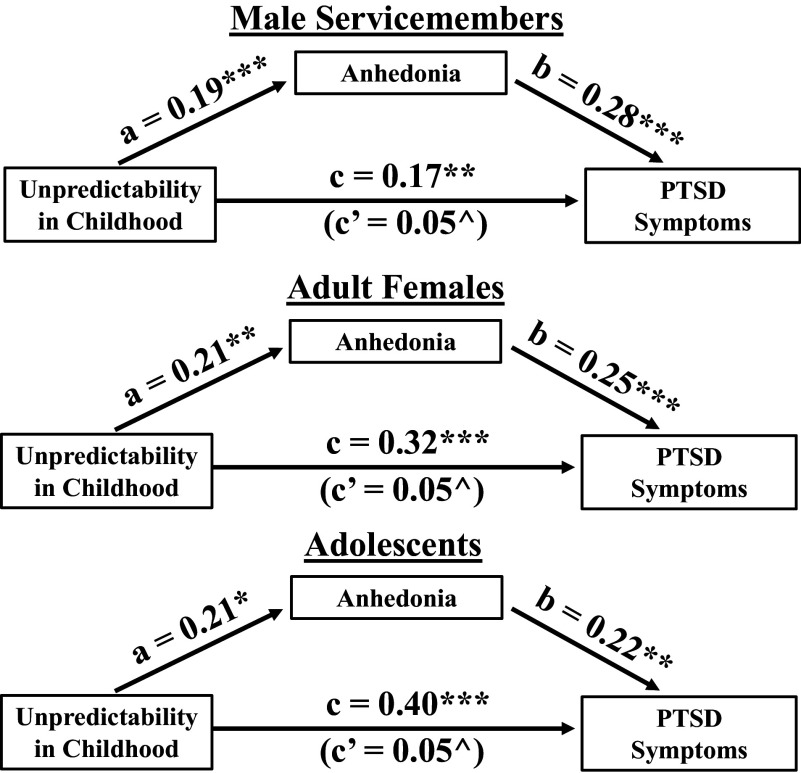
Mediation models depicting the effect of childhood unpredictability on later PTSD symptoms as mediated by anhedonia symptoms in service members (top), adult females (middle), and adolescents (bottom). Anhedonia symptoms were measured using the BDI-A for the service member cohort and using the MASQ-AD in the adult female and adolescent cohorts. PTSD symptoms were measured with the CAPS-IV in the service member cohort and with the PCL-5 in the adult females and adolescent cohorts. Unpredictability in childhood was measured with the QUIC for all three cohorts. C (direct effect) is the effect of childhood unpredictability on later PTSD symptoms not mediated by anhedonia symptoms, while c’ (indirect effect) is the effect of childhood unpredictability on later PTSD that is mediated by anhedonia symptoms. *Note:* QUIC = Questionnaire of Unpredictability in Childhood; BDI-A = Beck Depression Inventory Anhedonia Items; MASQ-AD = Mood and anxiety symptom questionnaire anhedonia subscale; CAPS-IV = Clinician Administered PTSD Scale for DSM-IV; PCL-5 = PTSD Checklist for DSM-5. ^bootstrapped 95% confidence interval does not contain zero; *p < .05; **p < .01; ***p < .001.

Mediational analyses revealed an indirect effect of childhood unpredictability on PTSD symptoms via higher anhedonia symptoms, which was significant in the male service member cohort (*b* = 0.055, 95% CI [0.02, 0.11]), the adult female cohort (*b* = 0.052, 95% CI [0.01, 0.13]), and the adolescent cohort (*b* = 0.046, 95% CI [0.01, 0.12]). Thus, in each cohort, a significant portion of the effect of childhood unpredictability on later PTSD symptoms was accounted for by higher anhedonia symptoms prior to the PTSD assessment. An illustration of the direct and indirect paths from unpredictability to PTSD symptoms for each cohort can be found in Figure 3.

### Sensitivity analyses

#### Consideration of childhood trauma

Results of regression models used to predict adult PTSD symptoms from QUIC and childhood LEC are shown in Table 2. In each cohort, higher childhood trauma was predictive of greater PTSD symptoms in adulthood (*p*s < .041). Critically, higher QUIC scores predicted higher PTSD symptoms even after covarying the effects of childhood trauma (*p*s < .001). After adjusting for childhood trauma, the indirect effect of QUIC on PTSD symptoms as mediated by anhedonia symptoms also remained significant in the male service member cohort (*b* = 0.052, 95% CI [0.020, 0.10]), the adult female cohort (*b* = 0.046, 95% CI = [0.007, 0.12]), and the adolescent cohort (*b* = 0.048, 95% CI [0.005, 0.12]). Thus, the relationship between childhood unpredictability and adult PTSD, as well as the mediating effect of anhedonia symptoms on this relationship, appeared to be unique from the effects of childhood trauma.

**Table 2.** Hierarchical regression models of childhood unpredictability predicting adult PTSD symptoms, controlling for childhood trauma

Cohort	DV	Step	Predictor	Step ΔR^2^	β (95% CI)	*t*	*p*
Service members	CAPS-IV total	1	Childhood LEC	.013	0.12 (0.005–0.23)	2.06	.041
		2	QUIC total	.040	0.21 (0.10–0.32)	3.68	<.001
Adult females	PCL–5 total	1	Childhood LEC	.070	0.27 (0.12–0.41)	3.61	<.001
		2	QUIC total	.101	0.33 (0.18–0.46)	4.56	<.001
Adolescents	PCL–5 total	1	Childhood LEC	.045	0.21 (0.05–0.37)	2.61	.010
		2	QUIC total	.166	0.43 (0.27–0.58)	5.51	<.001

*Note:* Childhood LEC was defined as the number of traumatic events endorsed as having directly experienced or witnessed prior to age 18 for the adult cohorts and as the number having directly experienced or witnessed concurrent with or prior to the age of QUIC administration for the adolescent cohort. QUIC = Questionnaire of Unpredictability in Childhood; LEC = Life events checklist; CAPS-IV = Clinician Administered PTSD Scale for DSM-IV; PCL-5 = PTSD Checklist for DSM-5.

#### Removing anhedonia-related symptoms from PTSD assessments

Removing anhedonia-related items from the PTSD symptom score did not alter the mediating effect of anhedonia symptoms, as its indirect effect remained significant in the male service member cohort (*b* = 0.047, 95% CI = [0.02, 0.10]), the adult female cohort (*b* = 0.055, 95% CI [0.01, 0.14]), and the adolescent cohort (*b* = 0.04, 95% CI [0.002, 0.09]). Thus, the mediating effect of anhedonia symptoms on the relationship between childhood unpredictability and PTSD symptoms did not appear to be driven by overlap with anhedonia-related content in PTSD assessment instruments.

#### Pre-deployment PTSD symptoms

Finally, within the male servicemember cohort, we reran the original mediation model including pre-deployment PTSD symptoms as a covariate. The indirect effect of QUIC via anhedonia symptoms remained significant, *b* = 0.019, 95% CI [0.0025, 0.048], suggesting that it was not an artifact of pre-existing PTSD symptoms.

## Discussion

The present study tested two hypotheses: (1) that childhood unpredictability acts as a risk factor for adult PTSD symptoms, and (2) that childhood unpredictability confers risk for adult PTSD in part via increased anhedonia symptoms. Overall, our findings were consistent with both hypotheses: Higher childhood unpredictability predicted greater adult PTSD symptoms across three different participant cohorts (male service members, adult females, and adolescents), and the effect of childhood unpredictability on adult PTSD was partially mediated by higher anhedonia symptoms in each cohort. Critically, the mediating effect of anhedonia symptoms remained significant in each cohort when covarying levels of childhood trauma and removing anhedonia-related symptoms from the PTSD outcome. Effects also remained significant in the male servicemember cohort when controlling for pre-deployment PTSD symptoms.

That childhood unpredictability predicted higher adult PTSD symptoms across three distinct longitudinal cohorts provides the strongest evidence to date that childhood unpredictability may act as a risk factor for adult PTSD. Although this association had been previously demonstrated in a cross-sectional investigation of Veterans (Spadoni et al., 2022), our study is the first to demonstrate a longitudinal association between childhood unpredictability and later PTSD, which provides more stringent evidence that a variable acts as a risk factor (Kraemer, 1997). Additionally, the fact that childhood trauma and childhood unpredictability each predicted significant, unique variance in adult PTSD symptoms helps broaden our understanding of the relationship between ELA and adult PTSD risk by demonstrating that such risk may not only be amplified by the experience of severe negative events during childhood (Breslau et al., 2014) but also by the degree to which parental interactions and the household environment in childhood are unpredictable.

Although our study cannot speak definitively as to *how* childhood unpredictability confers risk for adult PTSD, our mediation findings suggest that it may partly stem from the disruption of reward processes earlier in life. A variety of studies have demonstrated that unpredictability early in life can disrupt maturation of reward and stress systems (Baram et al., 2012; Davis et al., 2022; Davis et al., 2017; Granger et al., 2021). For instance, early life unpredictability in animals has been shown to reduce reward-seeking behavior in adulthood due, in part to the disruption of ventral striatum circuits that affect reward sensitivity, reward processing, and the motivational conflict between reward and threat (Levis et al., 2022; Birnie et al., 2020; Bolton et al., 2018). Although they cannot directly interrogate the health of these circuits, self-reported anhedonia symptoms are consistent with the existence of disrupted reward processing, suggesting that the relationship between childhood unpredictability and anhedonia symptoms in our study may have derived from a similar mechanism. Importantly, disruptions in the ventral striatum and its associated reward circuitry have also been implicated in PTSD (for reviews, see Vinograd, Stout, & Risbrough, 2022; Lokshina, Nickelsen, & Liberzon, 2021; Jia et al., 2023), and anhedonia symptoms have been shown to increase risk for the development of subsequent PTSD (Acheson et al., 2022). Taken together, these pieces of evidence are consistent with the hypothesis that early life unpredictability leads to increased anhedonia symptoms, which in turn confer risk for PTSD. Importantly, while there appears to be a particularly strong link between disrupted reward processing and anhedonia-related PTSD symptoms (Jia et al., 2023; Lokshina et al., 2021; Vinograd et al., 2022), the mediating effect of anhedonia symptoms in our study was unchanged when anhedonia-related content was removed from our PTSD assessments. Hence, the anhedonia-mediated effect of childhood unpredictability on PTSD cannot be explained as symptomatic overlap between PTSD and anhedonia, suggesting that it reflects a unique pathway that confers disruption in non-anhedonia-related PTSD symptoms.

The present study is not without limitations. First, only one cohort (male service members, N=317) included an earlier PTSD assessment that was concurrent with the anhedonia symptom assessment, which allowed us to test whether subsequent PTSD symptoms truly developed after anhedonia symptoms or were merely assessed after them. Second, the QUIC was administered close to a decade after childhood in the adult cohorts, rendering these participants susceptible to retrospective recall biases that could affect the validity of their scores. However, the fact that the QUIC was nonetheless associated with anhedonia symptoms that were assessed 10 years prior to the QUIC assessment suggests that its relations with anhedonia symptoms cannot be explained by state-dependent recall biases. Third, although we utilized self-reported anhedonia symptoms to make inferences about reward system functioning, the reward system is multifaceted, so its health can be interrogated in a variety of ways. For instance, behavioral paradigms that measure reinforcement learning and effort-based decision-making could index other aspects of the reward system that may be just as strong, if not stronger, mediators of the relationship between childhood unpredictability and PTSD. Finally, we did not test whether the pathway from childhood unpredictability through anhedonia symptoms might confer risk for other mental health issues besides PTSD. Indeed, cross-sectional data indicate that childhood unpredictability is also associated with greater anxiety disorder symptoms in adults and adolescents (Glynn et al., 2019; Spadoni et al., 2022), suggesting that a similar risk pathway may be at play for anxiety-related issues.

This study also possesses several notable strengths. Chief among these is the reliability of our findings despite methodological, demographic, and developmental differences across cohorts. In particular, the effect of childhood unpredictability on PTSD and the mediating effect of anhedonia symptoms on this relationship were very similar across cohorts despite utilizing different instruments to assess PTSD (i.e. PCL-5 versus CAPS-IV) and anhedonia symptoms (i.e. MASQ-AD versus BDI-A) and adhering to different assessment schedules (e.g. QUIC administered a few years before anhedonia/PTSD in adolescent and adult female cohorts versus 10 years after in the male servicemember cohort). Relatedly, the consistency of our findings across different populations not only provides further assurance that the relations between childhood unpredictability, anhedonia symptoms, and PTSD are real, but also that they are generalizable to people of different backgrounds (e.g. adolescents versus adults, males versus females, civilians versus service members, etc.).

Clinically, our findings imply that risk for PTSD could be partially mitigated through two distinct strategies: (1) restoring reward system functioning during adolescence or (2) making childhood more structured and predictable. Although both strategies could be effective, the latter approach may be preferable for several reasons. First, fostering more consistent routines and stable caregiving environments could support the normative maturation of reward circuitry, thereby addressing the proximal vulnerability for PTSD implicated in our findings. Indeed, engagement in structured caregiving practices (e.g. structured meal or bedtime routines, regular play or family time) has been found to buffer against the development of depressive symptoms in preschool children (Glynn et al., 2021; Davis & Glynn, 2024), consistent with animal models where predictable maternal care promoted healthy reward system development (Birnie et al., 2023; Bolton et al., 2018; Kangas et al., 2022; Levis et al., 2022). Second, making childhood more structured would also buffer against additional risk pathways that originate with childhood unpredictability, beyond that which occurs via reward system disruption. Such an assertion is consistent with our observation that anhedonia symptoms only partially mediated the relationship between childhood unpredictability and PTSD risk. Finally, promoting structure and predictability in daily routines represents a low-cost, non-invasive strategy that can be implemented within families or caregiving settings, especially when caregivers are provided with appropriate guidance and support. To this end, building awareness of the detrimental nature of childhood unpredictability among key stakeholders (e.g. parents, physicians) represents perhaps the most direct method for reducing its effects. Indeed, efforts are already underway to begin screening for unpredictable caregiving practices in pediatric settings so that caregivers are better equipped to guard against such practices in their daily lives (Glynn, 2025).

In conclusion, the present study found childhood unpredictability to be associated with greater adult PTSD symptoms and that this relationship is partially explained by higher anhedonia symptoms earlier in life. These findings implicate childhood unpredictability as a novel risk factor for adult PTSD and suggest that such risk may be conferred in part through the development of disrupted reward processes, which are consistent with the presence of higher anhedonia symptoms. Our results imply that risk for PTSD could be partially mitigated through making childhood more structured and predictable and possibly through interventions to enhance positive affect and reward processing in late adolescence/early adulthood (Davis & Glynn, 2024; Glynn et al., 2024). Future studies should more carefully track changes in childhood unpredictability, trauma, anhedonia symptoms, PTSD, and other variables over time and incorporate objective biological measures of stress reactivity and reward processing. Such work will help more firmly establish the mechanism through which childhood unpredictability confers risk for PTSD and allow us to develop more targeted interventions to reduce risk for PTSD.

## References

[r1] Acheson, D. T., Vinograd, M., Nievergelt, C. M., Yurgil, K. A., Moore, T. M., Risbrough, V. B., & Baker, D. G. (2022). Prospective examination of pre-trauma anhedonia as a risk factor for post-traumatic stress symptoms. European Journal of Psychotraumatology, 13(1), 2015949. 10.1080/20008198.2021.2015949. 35070161 PMC8774051

[r49] Admon, R., Milad, M. R., & Hendler, T. (2013). A causal model of post-traumatic stress disorder: Disentangling predisposed from acquired neural abnormalities. Trends in Cognitive Sciences, 17(7), 337–347. 10.1016/j.tics.2013.05.005. 23768722

[r2] Akiki, T. J., Jubeir, J., Bertrand, C., Tozzi, L., & Williams, L. M. (2025). Neural circuit basis of pathological anxiety. Nature Reviews Neuroscience, 26(1), 5–22. 10.1038/s41583-024-00880-4. 39604513

[r3] Aran, Ö., Swales, D. A., Bailey, N. A., Korja, R., Holmberg, E., Eskola, E., … Davis, E. P. (2024). Across ages and places: Unpredictability of maternal sensory signals and child internalizing behaviors. Journal of Affective Disorders, 347, 557–567. 10.1016/j.jad.2023.11.068. 38007106 PMC10843791

[r4] Baker, D., Nash, W., Litz, B., Geyer, M., Risbrough, V., & Nievergelt, C. (2012). Predictors of risk and resilience for posttraumatic stress disorder among ground combat marines: Methods of the marine resiliency study. Preventing Chronic Disease. 10.5888/pcd9.110134. PMC3431952 22575082

[r5] Baram, T. Z., Davis, E. P., Obenaus, A., Sandman, C. A., Small, S. L., Solodkin, A., & Stern, H. (2012). Fragmentation and unpredictability of early-life experience in mental disorders. American Journal of Psychiatry, 169(9), 907–915. 10.1176/appi.ajp.2012.11091347. 22885631 PMC3483144

[r50] Beck, A. T., Steer, R. A., & Brown, G. (1996). Beck depression inventory–II (BDI-II) [Database record]. *APA PsycTests*. 10.1037/t00742-000.

[r7] Bennett, M. M., Davis, K. E., & Fitzgerald, J. M. (2023). Neural correlates of reward processing in the onset, maintenance, and treatment of posttraumatic stress disorder. Biological Psychiatry: Cognitive Neuroscience and Neuroimaging, 8(9), 884–890. 10.1016/j.bpsc.2023.05.007. 37263417

[r8] Birnie, M. T., & Baram, T. Z. (2022). Principles of emotional brain circuit maturation. Science, 376(6597), 1055–1056. 10.1126/science.abn401. 35653483 PMC9840462

[r9] Birnie, M. T., Kooiker, C. L., Short, A. K., Bolton, J. L., Chen, Y., & Baram, T. Z. (2020). Plasticity of the reward circuitry after early-life adversity: Mechanisms and significance. Biological Psychiatry, 87(10), 875–884. 10.1016/j.biopsych.2019.12.018. 32081365 PMC7211119

[r10] Birnie, M. T., Short, A. K., De Carvalho, G. B., Taniguchi, L., Gunn, B. G., Pham, A. L., Itoga, C. A., Xu, X., Chen, L. Y., Mahler, S. V., Chen, Y., & Baram, T. Z. (2023). Stress-induced plasticity of a CRH/GABA projection disrupts reward behaviors in mice. Nature Communications, 14(1), 1088. 10.1038/s41467-023-36780-x. PMC9968307 36841826

[r11] Blake, D. D., Weathers, F. W., Nagy, L. M., Kaloupek, D. G., Gusman, F. D., Charney, D. S., & Keane, T. M. (1995). The development of a clinician-administered PTSD scale. Journal of Traumatic Stress, 8(1), 75–90. 10.1007/BF02105408. 7712061

[r12] Blevins, C. A., Weathers, F. W., Davis, M. T., Witte, T. K., & Domino, J. L. (2015). The posttraumatic stress disorder checklist for *DSM-5* (PCL-5): Development and initial psychometric evaluation. Journal of Traumatic Stress, 28(6), 489–498. 10.1002/jts.22059. 26606250

[r51] Bolton, J. L., Molet, J., Regev, L., Chen, Y., Rismanchi, N., Haddad, E., Yang, D. Z., Obenaus, A., & Baram, T. Z. (2018). Anhedonia following early-life adversity involves aberrant interaction of reward and anxiety circuits and is reversed by partial silencing of amygdala corticotropin-releasing hormone gene. Biological Psychiatry, 83(2), 137–147. 10.1016/j.biopsych.2017.08.023. 29033027 PMC5723546

[r14] Breslau, N., Koenen, K. C., Luo, Z., Agnew-Blais, J., Swanson, S., Houts, R. M., Poulton, R., & Moffitt, T. E. (2014). Childhood maltreatment, juvenile disorders and adult post-traumatic stress disorder: A prospective investigation. Psychological Medicine, 44(9), 1937–1945. 10.1017/S0033291713002651. 24168779 PMC4107193

[r52] Buckby, J. A., Yung, A. R., Cosgrave, E. M., & Killackey, E. J. (2007). Clinical utility of the Mood and Anxiety Symptom Questionnaire (MASQ) in a sample of young help-seekers. BMC Psychiatry, 7, 50. 10.1186/1471-244X-7-50. 17868477 PMC2151061

[r53] Clark, L. A., & Watson, D. (1991). Tripartite model of anxiety and depression: Psychometric evidence and taxonomic implications. Journal of Abnormal Psychology, 100(3), 316. 1918611 10.1037//0021-843x.100.3.316

[r54] Davis, E. P., & Glynn, L. M. (2024). Annual Research Review: The power of predictability–patterns of signals in early life shape neurodevelopment and mental health trajectories. Journal of Child Psychology and Psychiatry, 65(4), 508–534. 10.1111/jcpp.13958Di. 38374811 PMC11283837

[r15] Davis, E. P., Stout, S. A., Molet, J., Vegetabile, B., Glynn, L. M., Sandman, C. A., Heins, K., Stern, H., & Baram, T. Z. (2017). Exposure to unpredictable maternal sensory signals influences cognitive development across species. Proceedings of the National Academy of Sciences, 114(39), 10390–10395. 10.1073/pnas.1703444114. PMC5625898 28893979

[r16] Davis, E. P., McCormack, K., Arora, H., Sharpe, D., Short, A. K., Bachevalier, J., Glynn, L. M., Sandman, C. A., Stern, H. S., Sanchez, M., & Baram, T. Z. (2022). Early life exposure to unpredictable parental sensory signals shapes cognitive development across three species. Frontiers in Behavioral Neuroscience, 16, 960262. 10.3389/fnbeh.2022.960262. 36338881 PMC9630745

[r55] Der-Avakian, A., & Markou, A. (2012). The neurobiology of anhedonia and other reward-related deficits. Trends in Neurosciences, 35(1), 68–77. 10.1016/j.tins.2011.11.005. 22177980 PMC3253139

[r17] Ellis, B. J., Sheridan, M. A., Belsky, J., & McLaughlin, K. A. (2022). Why and how does early adversity influence development? Toward an integrated model of dimensions of environmental experience. Development and Psychopathology, 34, 447–471. 35285791 10.1017/S0954579421001838

[r56] Glynn, L. M. (2025). Predictability can reduce the burden of adverse childhood experiences: Policies to promote it. Policy Insights from the Behavioral and Brain Sciences, 12(1), 68–76. 10.1177/23727322241304354.

[r19] Glynn, L. M., & Baram, T. Z. (2019). The influence of unpredictable, fragmented parental signals on the developing brain. Frontiers in Neuroendocrinology, 53, 100736. 10.1016/j.yfrne.2019.01.002. 30711600 PMC6776465

[r20] Glynn, L. M., Stern, H. S., Howland, M. A., Risbrough, V. B., Baker, D. G., Nievergelt, C. M., Baram, T. Z., & Davis, E. P. (2019). Measuring novel antecedents of mental illness: The questionnaire of unpredictability in childhood. Neuropsychopharmacology, 44(5), 876–882. 10.1038/s41386-018-0280-9. 30470840 PMC6461958

[r21] Glynn, L. M., Davis, E. P., Luby, J. L., Baram, T. Z., & Sandman, C. A. (2021). A predictable home environment may protect child mental health during the COVID-19 pandemic. Neurobiology of Stress, 14, 100291. 10.1016/j.ynstr.2020.100291. 33532520 PMC7823041

[r22] Granger, S. J., Glynn, L. M., Sandman, C. A., Small, S. L., Obenaus, A., Keator, D. B., … Davis, E. P. (2021). Aberrant maturation of the uncinate fasciculus follows exposure to unpredictable patterns of maternal signals. Journal of Neuroscience, 41(6), 1242–1250. 10.1523/JNEUROSCI.0374-20.2020. 33328295 PMC7888232

[r23] Haim-Nachum, S., Amsalem, D., Lazarov, A., Zabag, R., Neria, Y., & Sopp, M. R. (2024). Anhedonia mediates the relationships between childhood trauma and symptom severity of PTSD and depression, but not of social anxiety. Journal of Affective Disorders, 344, 577–584. 10.1016/j.jad.2023.10.107. 37863363

[r24] Hunt, C., Vinograd, M., Glynn, L. M., Davis, E. P., Baram, T. Z., Stern, H., Nievergelt, C., Cuccurazzu, B., Napan, C., Delmar, D., Baker, D. G., & Risbrough, V. B. (2024). Childhood unpredictability is associated with increased risk for long- and short-term depression and anhedonia symptoms following combat deployment. Journal of Mood & Anxiety Disorders, 6, 100045. 10.1016/j.xjmad.2023.100045. 38911511 PMC11192232

[r25] Jia, R., Ruderman, L., Pietrzak, R. H., Gordon, C., Ehrlich, D., Horvath, M., … Levy, I. (2023). Neural valuation of rewards and punishments in posttraumatic stress disorder: A computational approach. Translational Psychiatry, 13(1), 101. 10.1038/s41398-023-02388-4. 36977676 PMC10050320

[r28] Kalisch, R., Russo, S. J., & Müller, M. B. (2024). Neurobiology and systems biology of stress resilience. Physiological Reviews, 104(3), 1205–1263. 10.1152/physrev.00042.2023. 38483288 PMC11381009

[r29] Kangas, B. D., Short, A. K., Luc, O. T., Stern, H. S., Baram, T. Z., & Pizzagalli, D. A. (2022). A cross-species assay demonstrates that reward responsiveness is enduringly impacted by adverse, unpredictable early-life experiences. Neuropsychopharmacology, 47(3), 767–775. 10.1038/s41386-021-01250-9. 34921225 PMC8682039

[r57] Keen, R., Chen, J. T., Slopen, N., Sandel, M., Copeland, W. E., & Tiemeier, H. (2023). Prospective associations of childhood housing insecurity with anxiety and depression symptoms during childhood and adulthood. JAMA Pediatrics, 177(8), 818–826. 10.1001/jamapediatrics.2023.1733. 37338896 PMC10282957

[r30] Kessler, R. C., Aguilar-Gaxiola, S., Alonso, J., Benjet, C., Bromet, E. J., Cardoso, G., Degenhardt, L., De Girolamo, G., Dinolova, R. V., Ferry, F., Florescu, S., Gureje, O., Haro, J. M., Huang, Y., Karam, E. G., Kawakami, N., Lee, S., Lepine, J.-P., Levinson, D., … Koenen, K. C. (2017). Trauma and PTSD in the WHO world mental health surveys. European Journal of Psychotraumatology, 8(sup5), 1353383. 10.1080/20008198.2017.1353383. 29075426 PMC5632781

[r31] Köster, M., Kayhan, E., Langeloh, M., & Hoehl, S. (2020). Making sense of the world: Infant learning from a predictive processing perspective. Perspectives on Psychological Science, 15(3), 562–571. 10.1177/1745691619895. 32167407 PMC7243078

[r32] Kraemer, H. C. (1997). Coming to terms with the terms of risk. Archives of General Psychiatry, 54(4), 337. 10.1001/archpsyc.1997.01830160065009. 9107150

[r33] Levis, S. C., Birnie, M. T., Bolton, J. L., Perrone, C. R., Montesinos, J. S., Baram, T. Z., & Mahler, S. V. (2022). Enduring disruption of reward and stress circuit activities by early-life adversity in male rats. Translational Psychiatry, 12(1), 251. 10.1038/s41398-022-01988-w. 35705547 PMC9200783

[r34] Lokshina, Y., Nickelsen, T., & Liberzon, I. (2021). Reward processing and circuit dysregulation in posttraumatic stress disorder. Frontiers in Psychiatry, 12, 559401. 10.3389/fpsyt.2021.559401. 34122157 PMC8193060

[r35] MacKinnon, D. P., Lockwood, C. M., Hoffman, J. M., West, S. G., & Sheets, V. (2002). A comparison of methods to test mediation and other intervening variable effects. Psychological Methods, 7(1), 83–104. 10.1037/1082-989X.7.1.83. 11928892 PMC2819363

[r36] Marsh, S., Dobson, R., & Maddison, R. (2020). The relationship between household chaos and child, parent, and family outcomes: A systematic scoping review. BMC Public Health, 20(1), 513. 10.1186/s12889-020-08587-8. 32316937 PMC7175577

[r58] Naragon-Gainey, K., Watson, D., & Markon, K. E. (2009). Differential relations of depression and social anxiety symptoms to the facets of extraversion/positive emotionality. Journal of Abnormal Psychology, 118(2), 299–310. 10.1037/a0015637. 19413405 PMC2794796

[r59] Null, K. E., Duda, J. M., & Pizzagalli, D. A. (2024). Social support mediates the effects of childhood unpredictability on anhedonia: A retrospective investigation in an online adult community sample. Journal of Mood & Anxiety Disorders, 6, 100057. 10.1016/j.xjmad.2024.100057. 40655919 PMC12243996

[r60] Pizzagalli, D. A. (2014). Depression, stress, and anhedonia: Toward a synthesis and integrated model. Annual Review of Clinical Psychology, 10(1), 393–423. 10.1146/annurev-clinpsy-050212-185606. PMC3972338 24471371

[r38] Preacher, K. J., & Hayes, A. F. (2004). SPSS and SAS procedures for estimating indirect effects in simple mediation models. Behavior Research Methods, Instruments, & Computers, 36(4), 717–731. 10.3758/BF03206553. 15641418

[r39] Risbrough, V. B., Glynn, L. M., Davis, E. P., Sandman, C. A., Obenaus, A., Stern, H. S., Keator, D. B., Yassa, M. A., Baram, T. Z., & Baker, D. G. (2018). Does anhedonia presage increased risk of posttraumatic stress disorder?: Adolescent anhedonia and posttraumatic disorders. In E. Vermetten, D. G. Baker, & V. B. Risbrough (Eds.), Behavioral neurobiology of PTSD (Vol. 38, pp. 249–265). Springer International Publishing. 10.1007/7854_2018_51. PMC9167566 29796839

[r40] Short, A. K., & Baram, T. Z. (2019). Early-life adversity and neurological disease: Age-old questions and novel answers. Nature Reviews Neurology, 15(11), 657–669. 10.1038/s41582-019-0246-5. 31530940 PMC7261498

[r41] Spadoni, A. D., Vinograd, M., Cuccurazzu, B., Torres, K., Glynn, L. M., Davis, E. P., Baram, T. Z., Baker, D. G., Nievergelt, C. M., & Risbrough, V. B. (2022). Contribution of early-life unpredictability to neuropsychiatric symptom patterns in adulthood. Depression and Anxiety, 39(10–11), 706–717. 10.1002/da.23277. 35833573 PMC9881456

[r42] Sroufe, L. A. (2005). Attachment and development: A prospective, longitudinal study from birth to adulthood. Attachment & Human Development, 7, 349–367. 16332580 10.1080/14616730500365928

[r43] Stevens, J. S., Harnett, N. G., Lebois, L. A., Van Rooij, S. J., Ely, T. D., Roeckner, A., … Ressler, K. J. (2021). Brain-based biotypes of psychiatric vulnerability in the acute aftermath of trauma. American Journal of Psychiatry, 178(11), 1037–1049. 10.1176/appi.ajp.2021.20101526. 34645277 PMC9069566

[r61] Talkovsky, A. M., & Norton, P. J. (2015). The Mood and Anxiety Symptom Questionnaire across four ethnoracial groups in an undergraduate sample. American Journal of Orthopsychiatry, 85(5), 431. 10.1037/ort0000095. 26460703

[r62] Treadway, M. T., & Zald, D. H. (2011). Reconsidering anhedonia in depression: Lessons from translational neuroscience. Neuroscience & Biobehavioral Reviews, 35(3), 537–555. 10.1016/j.neubiorev.2010.06.006. 20603146 PMC3005986

[r44] Vinograd, M., Stout, D. M., & Risbrough, V. B. (2022). Anhedonia in posttraumatic stress disorder: Prevalence, phenotypes, and neural circuitry. In D. A. Pizzagalli (Ed.), Anhedonia: Preclinical, translational, and clinical integration (Vol. 58, pp. 185–199). Springer International Publishing. 10.1007/7854_2021_292. 34907507

[r201] von Stumm, S. (2024). Adolescents’ perceptions of household chaos predict their adult mental health: A twin-difference longitudinal cohort study. Psychological Science, 35(7), 736–748. 10.1177/09567976241242. 38717488 PMC13020938

[r63] Wang, Z., Cao, X., Zheng, X., Chen, Y., & Zhu, J. (2024). Abnormalities in brain structure following childhood unpredictability: A mechanism underlying depressive and anxiety symptoms. Psychological Medicine, 54(2), 299–307. 10.1017/S0033291723001526. 37264828

[r46] Weathers, F. W., Blake, D. D., Schnurr, P. P., Kaloupek, D. G., Marx, B. P., & Keane, T. M. (2013). Life Events Checklist for DSM-5 (LEC-5). National Center for PTSD. https://www.ptsd.va.gov.

[r47] Weathers, F. W., Litz, B. T., Keane, T. M., Palmieri, P. A., Marx, B. P., & Schnurr, P. P. (2013). PTSD Checklist for DSM-5 (PCL-5). National Center for PTSD. https://www.ptsd.va.gov.

[r64] Whitton, A. E., Treadway, M. T., & Pizzagalli, D. A. (2015). Reward processing dysfunction in major depression, bipolar disorder and schizophrenia. Current Opinion in Psychiatry, 28(1), 7–12. 10.1097/YCO.0000000000000122. 25415499 PMC4277233

[r48] Yurgil, K. A., Barkauskas, D. A., Vasterling, J. J., Nievergelt, C. M., Larson, G. E., Schork, N. J., Litz, B. T., Nash, W. P., & Baker, D. G. (2014). Association between traumatic brain injury and risk of posttraumatic stress disorder in active-duty marines. JAMA Psychiatry, 71(2), 149. 10.1001/jamapsychiatry.2013.3080. 24337530

